# The genome sequence of the black clock beetle, *Pterostichus madidus *(Fabricius, 1775)

**DOI:** 10.12688/wellcomeopenres.17347.1

**Published:** 2021-11-08

**Authors:** Liam M. Crowley, University of Oxford and Wytham Woods Genome Acquisition Lab, Darwin Tree of Life Barcoding collective, Wellcome Sanger Institute Tree of Life programme, Wellcome Sanger Institute Scientific Operations: DNA Pipelines collective, Tree of Life Core Informatics collective, Darwin Tree of Life Consortium

**Affiliations:** 1 Department of Zoology, University of Oxford, Oxford, UK

**Keywords:** Pterostichus madidus, black clock beetle, genome sequence, chromosomal

## Abstract

We present a genome assembly from an individual female *Pterostichus madidus *(the black clock beetle; Arthropoda; Insecta; Coleoptera; Carabidae). The genome sequence is 705 megabases in span. The majority (99.96%) of the assembly is scaffolded into 19 chromosomal pseudomolecules, with the X sex chromosome assembled.

## Species taxonomy

Eukaryota; Metazoa; Ecdysozoa; Arthropoda; Hexapoda; Insecta; Pterygota; Neoptera; Endopterygota; Coleoptera; Adephaga; Caraboidea; Carabidae; Harpalinae; Pterostichini; Pterostichus; Steropus; *Pterostichus madidus* (Fabricius, 1775) (NCBI:txid767470).

## Background

The black clock beetle, *Pterostichus madidus*, is a large, common species of ground beetle. It occurs across western and northern Europe and in the UK it is the most frequently recorded beetle in the family Carabidae. It can be found throughout a wide range of habitats where it is active during both the night and day. It is a relatively large (13-18 mm), black carabid with smoothly rounded pronotal hind angles. There are two subspecies, *Pterostichus madidus validus* Dejean, 1828, which has black femora, and *Pterostichus madidus concinnus* (Sturm, 1818), which has distinctive ‘wine red’ femora. *Pterostichus madidus* is omnivorous, being a predator and scavenger, but also feeding on plant material ( Luff, 1974). It is predominantly an annual species, laying eggs in late summer/autumn and larvae developing over the winter ( Luff & Others, 1973). Overwintered adults are active from spring/early summer and some adults, particularly at higher altitudes, are biennial ( Butterfield, 1996).

## Genome sequence report

**Figure 1.**
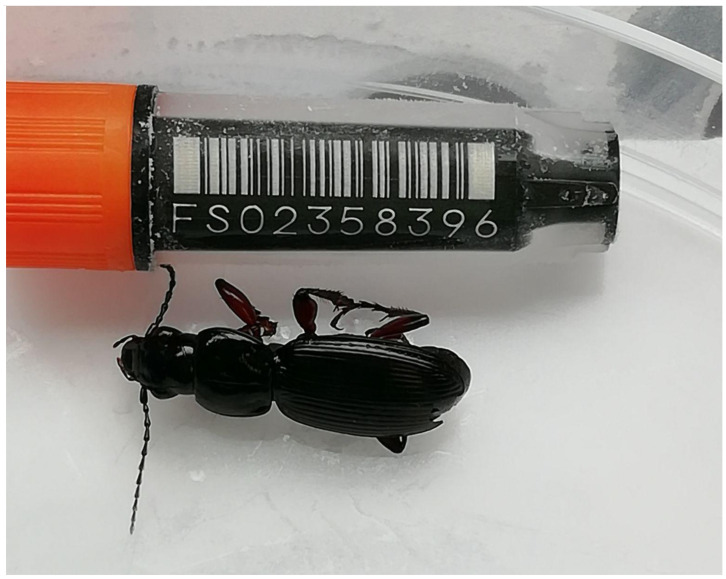
An image of the sequenced specimen, icPteMadi1, captured immediately prior to processing and preservation.

The genome was sequenced from one female *P. madidus* collected from Wytham Woods, Oxfordshire (biological vice-county: Berkshire), UK (latitude 51.775, longitude -1.326) ( Figure 1). A total of 34-fold coverage in Pacific Biosciences single-molecule long reads and 53-fold coverage in 10X Genomics read clouds were generated. Primary assembly contigs were scaffolded with chromosome conformation Hi-C data. Manual assembly curation corrected 142 missing/misjoins and removed 6 haplotypic duplications, reducing the assembly length by 0.18% and the scaffold number by 80.00%, and increasing the scaffold N50 by 58.29%.

**Figure 2.**
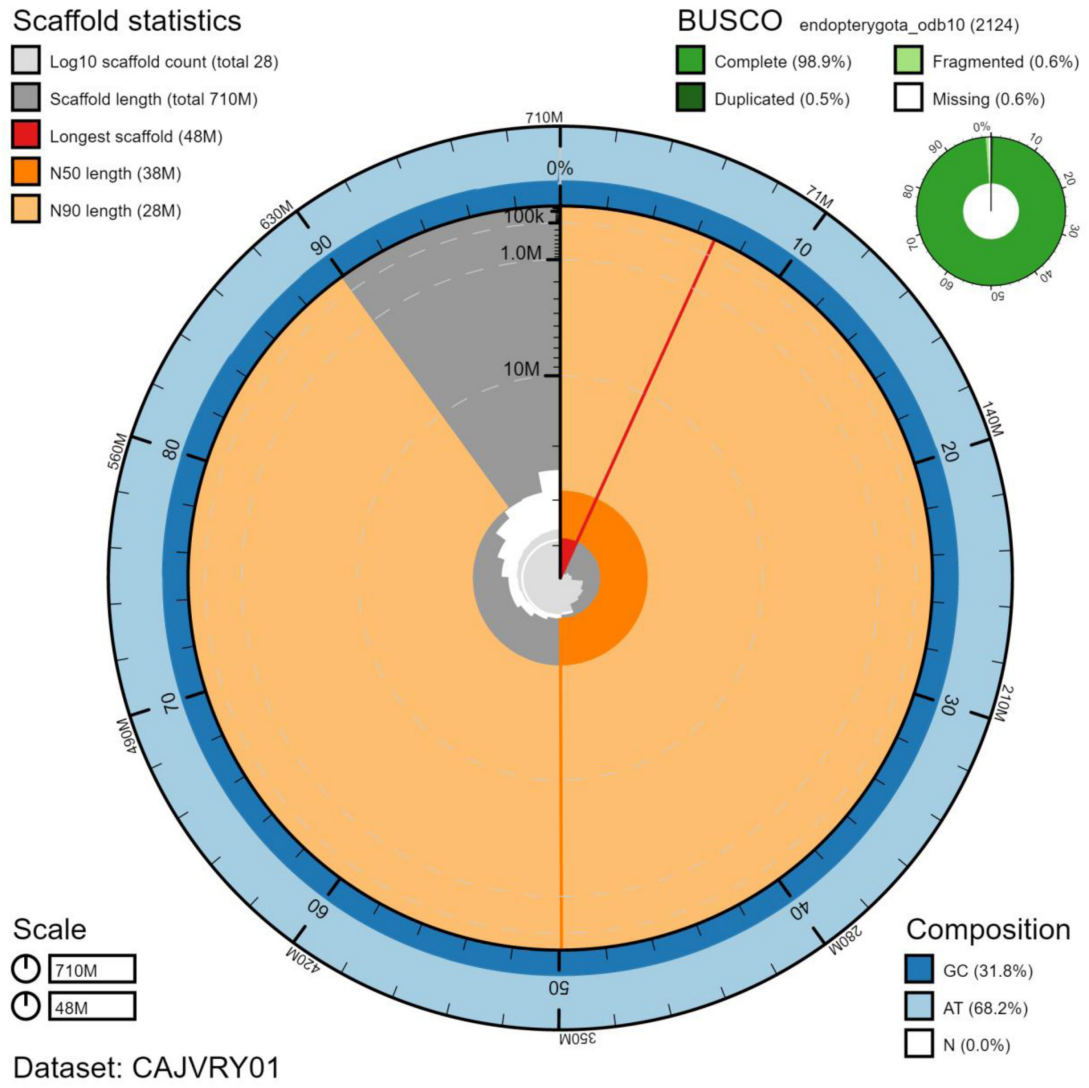
Genome assembly of *Pterostichus madidus*, icPteMadi1.1: metrics. The BlobToolKit Snailplot shows N50 metrics and BUSCO gene completeness. The main plot is divided into 1,000 size-ordered bins around the circumference with each bin representing 0.1% of the 705,160,476 bp assembly. The distribution of scaffold lengths is shown in dark grey with the plot radius scaled to the longest scaffold present in the assembly (47,997,105 bp, shown in red). Orange and pale-orange arcs show the N50 and N90 scaffold lengths (37,879,541 and 28,091,952 bp), respectively. The pale grey spiral shows the cumulative scaffold count on a log scale with white scale lines showing successive orders of magnitude. The blue and pale-blue area around the outside of the plot shows the distribution of GC, AT and N percentages in the same bins as the inner plot. A summary of complete, fragmented, duplicated and missing BUSCO genes in the endopterygota_odb10 set is shown in the top right. An interactive version of this figure is available at https://blobtoolkit.genomehubs.org/view/icPteMadi1.1/dataset/CAJVRY01/snail.

The final assembly has a total length of 705 Mb in 27 sequence scaffolds with a scaffold N50 of 37.9 Mb ( Table 1). The majority, 99.96%, of the assembly sequence was assigned to 19 chromosomal-level scaffolds, representing 18 autosomes (numbered by sequence length), and the X sex chromosome ( Figure 2– Figure 5; Table 2). Some regions of the genome have large repeats with less certain structure than the rest of the assembly, most notably chromosomes 14, 15 and 18. Chromosome 14 from 23.8 Mb onwards has strong Hi-C association with chromosome 18. The assembly has a BUSCO v5.1.2 ( Manni *et al*., 2021) completeness of 98.9% (single 98.4%, duplicated 0.5%) using the endopterygota_odb10 reference set. While not fully phased, the assembly deposited is of one haplotype. Contigs corresponding to the second haplotype have also been deposited.

**Table 1.** Genome data for *Pterostichus madidus*, icPteMadi1.1.

*Project accession data*	
Assembly identifier	icPteMadi1.1
Species	*Pterostichus madidus*
Specimen	icPteMadi1
NCBI taxonomy ID	NCBI:txid767470
BioProject	PRJEB45192
BioSample ID	SAMEA7520318
Isolate information	Female, head/thorax (genome assembly), abdomen (Hi-C)
*Raw data accessions*	
PacificBiosciences SEQUEL II	ERR6606793
10X Genomics Illumina	ERR6054945-ERR6054948
Hi-C Illumina	ERR6054949
*Genome assembly*	
Assembly accession	GCA_911728475.1
Accession of alternate haplotype	GCA_911728425.1
Span (Mb)	705
Number of contigs	184
Contig N50 length (Mb)	15.8
Number of scaffolds	27
Scaffold N50 length (Mb)	37.9
Longest scaffold (Mb)	48.0
BUSCO * genome score	C:98.9%[S:98.4%,D:0.5%],F:0.6%,M:0.6%,n:2124

*BUSCO scores based on the endopterygota_odb10 BUSCO set using v5.1.2. C= complete [S= single copy, D=duplicated], F=fragmented, M=missing, n=number of orthologues in comparison. A full set of BUSCO scores is available at https://blobtoolkit.genomehubs.org/view/icPteMadi1.1/dataset/CAJVRY01/busco.

**Figure 3.**
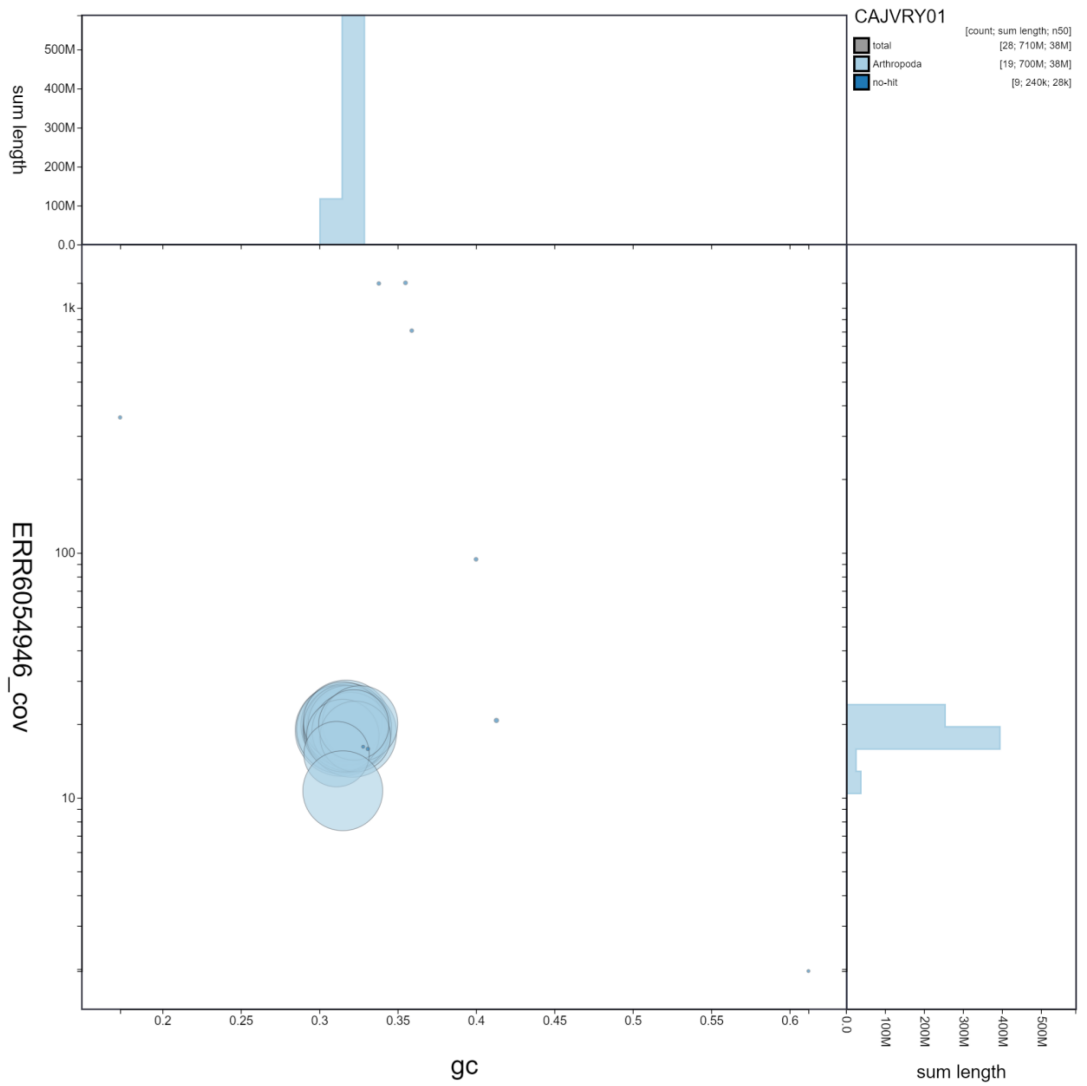
Genome assembly of *Pterostichus madidus*, icPteMadi1.1: GC coverage. BlobToolKit GC-coverage plot. Scaffolds are coloured by phylum. Circles are sized in proportion to scaffold length Histograms show the distribution of scaffold length sum along each axis. An interactive version of this figure is available at https://blobtoolkit.genomehubs.org/view/icPteMadi1.1/dataset/CAJVRY01/blob.

**Figure 4.**
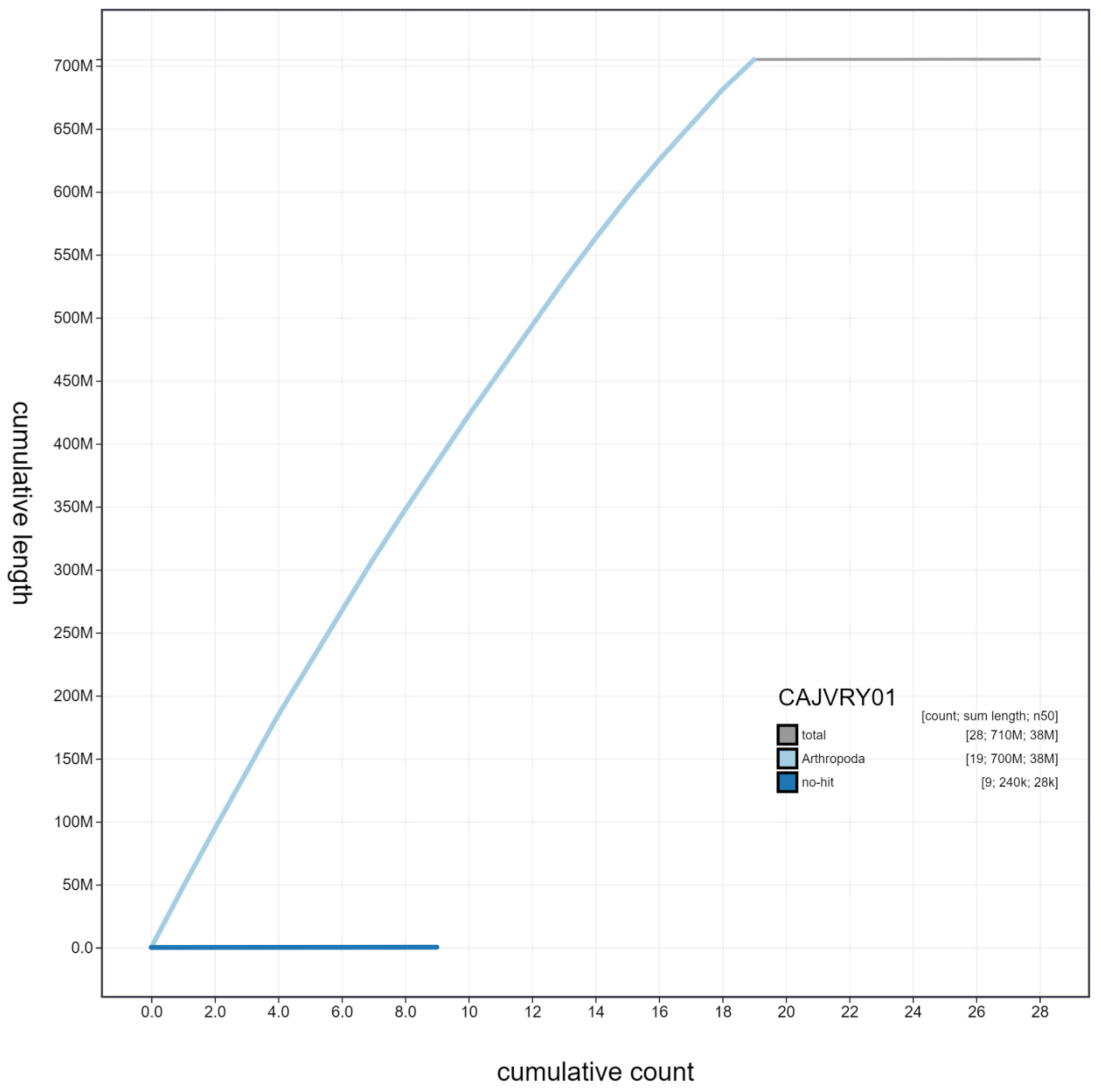
Genome assembly of *Pterostichus madidus*, icPteMadi1.1: cumulative sequence. BlobToolKit cumulative sequence plot. The grey line shows cumulative length for all scaffolds. Coloured lines show cumulative lengths of scaffolds assigned to each phylum using the buscogenes taxrule. An interactive version of this figure is available at https://blobtoolkit.genomehubs.org/view/icPteMadi1.1/dataset/CAJVRY01/cumulative.

**Figure 5.**
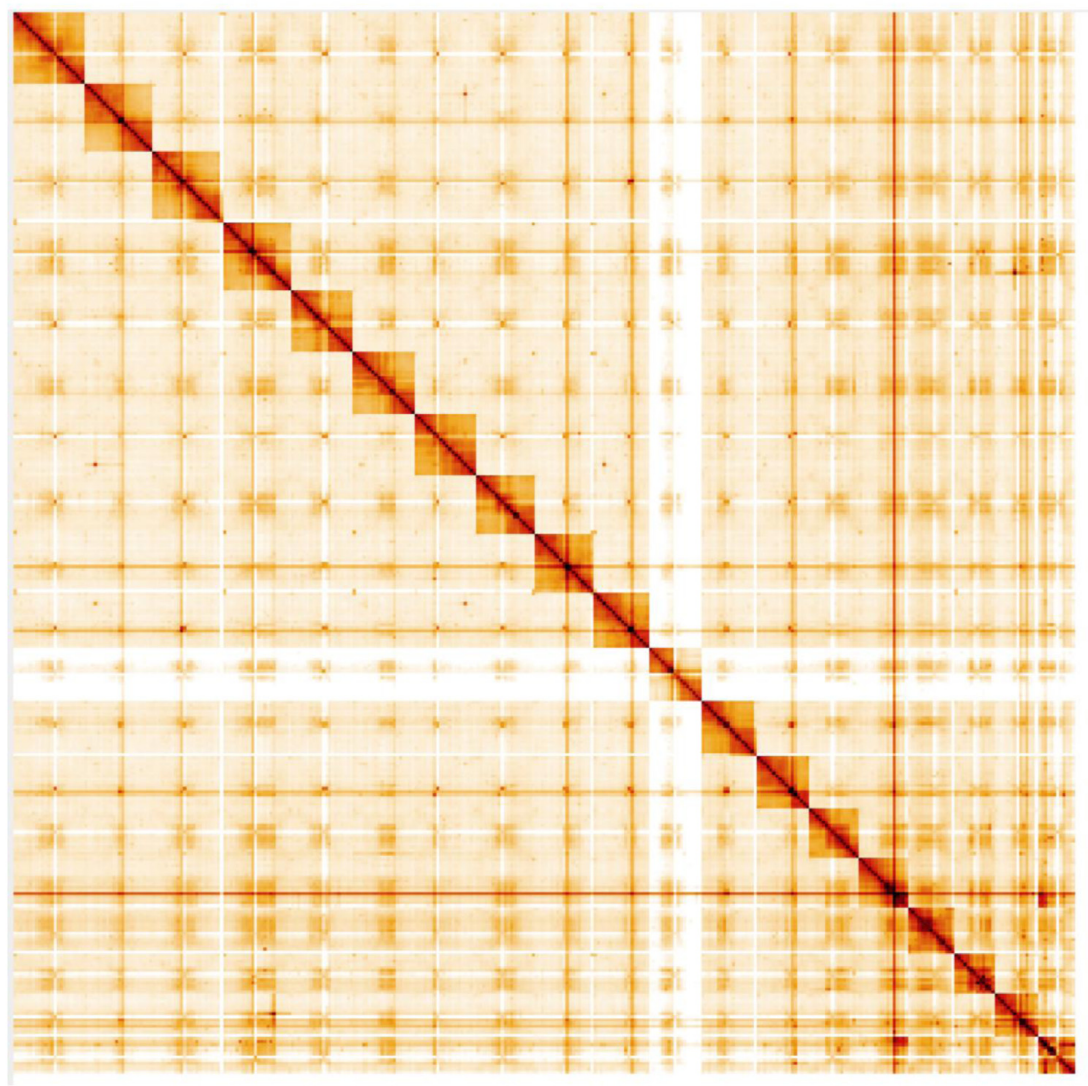
Genome assembly of *Pterostichus madidus*, icPteMadi1.1: Hi-C contact map. Hi-C contact map of the icPteMadi1.1 assembly, visualised in HiGlass.

**Table 2.** Chromosomal pseudomolecules in the genome assembly of *Pterostichus madidus*, icPteMadi1.1.

INSDC accession	Chromosome	Size (Mb)	GC%
OU452301.1	1	48.00	31.4
OU452302.1	2	46.20	31.7
OU452303.1	3	45.37	31.4
OU452304.1	4	44.84	32.1
OU452305.1	5	42.01	31.7
OU452306.1	6	41.31	31.9
OU452307.1	7	40.83	31.7
OU452308.1	8	38.74	31.8
OU452309.1	9	37.88	31.8
OU452310.1	10	37.21	31.6
OU452312.1	11	35.34	31.8
OU452313.1	12	35.33	31.5
OU452314.1	13	33.82	32
OU452315.1	14	32.21	32.6
OU452316.1	15	29.68	31.5
OU452317.1	16	28.09	32.3
OU452318.1	17	27.89	32.2
OU452319.1	18	23.99	31.1
OU452311.1	X	36.19	31.5
OU452320.1	MT	0.02	17.3
-	Unplaced	0.22	38.4

## Methods

### Sample acquisition, DNA extraction and sequencing

A single female *P. madidus* was collected from Wytham Woods, Oxfordshire (biological vice-county: Berkshire), UK (latitude 51.775, longitude -1.326) by Liam Crowley, University of Oxford, using a pooter. The sample was identified by the same individual, snap-frozen on dry ice and stored using a CoolRack.

DNA was extracted from the head/thorax tissue of *P. madidus* (icPteMadi1) at the Wellcome Sanger Institute (WSI) Scientific Operations core from the whole organism using the Qiagen MagAttract HMW DNA kit, according to the manufacturer’s instructions. Pacific Biosciences HiFi circular consensus and 10X Genomics read cloud DNA sequencing libraries were constructed according to the manufacturers’ instructions. Sequencing was performed by the Scientific Operations core at the WSI on Pacific Biosciences SEQUEL II and Illumina HiSeq X instruments. Hi-C data were generated from abdomen tissue of icPteMadi1 using the Arima v2 Hi-C kit and sequenced on an Illumina NovaSeq 6000 instrument.

### Genome assembly

Assembly was carried out with Hifiasm ( Cheng *et al*., 2021); haplotypic duplication was identified and removed with purge_dups ( Guan *et al*., 2020). One round of polishing was performed by aligning 10X Genomics read data to the assembly with longranger align, calling variants with freebayes ( Garrison & Marth, 2012). The assembly was then scaffolded with Hi-C data ( Rao *et al*., 2014) using SALSA2 ( Ghurye *et al*., 2019). The assembly was checked for contamination and corrected using the gEVAL system ( Chow *et al*., 2016) as described previously ( Howe *et al*., 2021). Manual curation ( Howe *et al*., 2021) was performed using gEVAL, HiGlass ( Kerpedjiev *et al*., 2018) and Pretext. The mitochondrial genome was assembled using MitoHiFi ( Uliano-Silva *et al*., 2021). The genome was analysed and BUSCO scores generated within the BlobToolKit environment ( Challis *et al*., 2020). Table 3 contains a list of all software tool versions used, where appropriate.

**Table 3.** Software tools used.

Software tool	Version	Source
Hifiasm	0.14-r312	Cheng *et al.,* 2021
purge_dups	1.2.3	Guan *et al.,* 2020
SALSA2	2.2	Ghurye *et al.,* 2019
longranger align	2.2.2	https://support.10xgenomics.com/genome-exome/software/pipelines/latest/advanced/other-pipelines
freebayes	1.3.1-17-gaa2ace8	Garrison & Marth, 2012
MitoHiFi	2.11.3	Uliano-Silva *et al.,* 2021
gEVAL	N/A	Chow *et al.,* 2016
HiGlass	1.11.6	Kerpedjiev *et al.,* 2018
PretextView	0.2.x	https://github.com/wtsi-hpag/PretextView
BlobToolKit	2.6.2	Challis *et al.,* 2020

### Ethics/compliance issues

The materials that have contributed to this genome note have been supplied by a Darwin Tree of Life Partner. The submission of materials by a Darwin Tree of Life Partner is subject to the Darwin Tree of Life Project Sampling Code of Practice. By agreeing with and signing up to the Sampling Code of Practice, the Darwin Tree of Life Partner agrees they will meet the legal and ethical requirements and standards set out within this document in respect of all samples acquired for, and supplied to, the Darwin Tree of Life Project. Each transfer of samples is further undertaken according to a Research Collaboration Agreement or Material Transfer Agreement entered into by the Darwin Tree of Life Partner, Genome Research Limited (operating as the Wellcome Sanger Institute), and in some circumstances other Darwin Tree of Life collaborators.

## Data availability

European Nucleotide Archive: Pterostichus madidus (black clock beetle). Accession number PRJEB45192; https://identifiers.org/ena.embl/PRJEB45192.

The genome sequence is released openly for reuse. The *P. madidus* genome sequencing initiative is part of the Darwin Tree of Life (DToL) project. All raw sequence data and the assembly have been deposited in INSDC databases. The genome will be annotated and presented through the Ensembl pipeline at the European Bioinformatics Institute. Raw data and assembly accession identifiers are reported in Table 1.

## Author information

Members of the University of Oxford and Wytham Woods Genome Acquisition Lab are listed here: https://doi.org/10.5281/zenodo.4789929.

Members of the Darwin Tree of Life Barcoding collective are listed here: https://doi.org/10.5281/zenodo.4893704.

Members of the Wellcome Sanger Institute Tree of Life programme collective are listed here: https://doi.org/10.5281/zenodo.5377053.

Members of Wellcome Sanger Institute Scientific Operations: DNA Pipelines collective are listed here: https://doi.org/10.5281/zenodo.4790456.

Members of the Tree of Life Core Informatics collective are listed here: https://doi.org/10.5281/zenodo.5013542.

Members of the Darwin Tree of Life Consortium are listed here: https://doi.org/10.5281/zenodo.4783559.
