# Supplementary material for: The genome sequence of the black clock beetle, Pterostichus madidus (Fabricius, 1775)
Source: Wellcome Open Res. 2021 Nov 8;6:301. [Version 1] doi: 10.12688/wellcomeopenres.17347.1 (PMC9315234; doi:10.12688/wellcomeopenres.17347.1)
Supplement: Peer review history

# Peer review: Reviewer response for version 1

**DOI:** 10.21956/wellcomeopenres.19177.r51468

**Published:** 2022-07-25

**Authors:** Lukas Zangl

**Affiliations:** 1 Institute of Biology, University of Graz, Graz, Austria

In this study, the authors provide a full length chromosome-level quality genome of the ground beetle Pterostichus madidus. The genome was composed of PacBio long-reads as well as 10X Genomics Illumina short-read data. High BUSCO scores suggest high levels of coding gene completeness and homogenous GC values as well as low numbers of non-traget scaffolds indicate low levels of contamination.

 All raw data as well as assembly and annotation are publicly available and the methods and tools described will allow for good reproducibility. My only remark in this context regards the manual edits, which are mentioned, but not described in further detail. Maybe there are some comments on rationale and procedure that would be appropriate/needed to insure full reproducibility.

 Furthermore, information about the two subspecies is given, however the authors missed to provide a comment on which subspecies they have actually sequenced.

 Finally, despite the apparent good quality of the assembled and annotated genome, I had a hard time interpreting the Snailplot (specifically the grey and orange parts), however, I have to admit that I am not too familiar with his kind of representation. Therefore, I would suggest adding a few comments in the main text about what can be seen in order to increase clarification for all readers in general.

Are sufficient details of methods and materials provided to allow replication by others?

Yes

Is the rationale for creating the dataset(s) clearly described?

Yes

Are the datasets clearly presented in a useable and accessible format?

Yes

Are the protocols appropriate and is the work technically sound?

Yes

Reviewer Expertise:

zoology, biodiversity research, genetics (inlcuding some genomics)

I confirm that I have read this submission and believe that I have an appropriate level of expertise to confirm that it is of an acceptable scientific standard.

# Peer review: Reviewer response for version 1

**DOI:** 10.21956/wellcomeopenres.19177.r46962

**Published:** 2021-12-20

**Authors:** James Pflug

**Affiliations:** 1 Department of Integrative Biology, Oregon State University, Corvallis, OR, USA

Summary:

 This paper reports on the successful sequencing of the genome of the ground beetle *Pterostichus madidus*. The authors employed PacBio and 10X Genomics sequencing to produce a 705 Mb assembly. The authors also performed Hi-C experiments, with the data suggesting that the genome is divided into 19 chromosomal pseudomolecules. The assembly contained matches to the vast majority of BUSCO endopterygota_odb10 reference genes, suggesting that the coding gene content of the assembly is relatively complete.  

 The paper provides a clear justification for the purpose of the study, as well as the methods and means used to obtain the data. The resulting genome assembly appears to be relatively high quality based on the analyses performed. All relevant data have been deposited in the appropriate locations and are readily accessible. Overall, the experiments conducted are sound, and the manuscript is well written and appropriate for the Data Note format.  

 General Comments:

- Has *P. madidus*'s genome size (or that of a closely related species) been established via flow cytography or other fluorometric methods? If so, how does it compare to the 705 Mb assembly?
- Figure 2 (i.e., the BlobToolKit Snailplot) is somewhat difficult for me to understand at first look. In particular, the orange and grey histogram section in the center of the large circle. I'm aware this style of plot is fairly common, and others who are more familiar with snailplots will not have trouble interpreting them, but readers like me would appreciate some additional clarification of what's going on.

Are sufficient details of methods and materials provided to allow replication by others?

Yes

Is the rationale for creating the dataset(s) clearly described?

Yes

Are the datasets clearly presented in a useable and accessible format?

Yes

Are the protocols appropriate and is the work technically sound?

Yes

Reviewer Expertise:

Genomics and Systematics of the beetle family Carabidae

I confirm that I have read this submission and believe that I have an appropriate level of expertise to confirm that it is of an acceptable scientific standard.
